# Hyperactivation is sufficient to release porcine sperm from immobilized oviduct glycans

**DOI:** 10.1038/s41598-022-10390-x

**Published:** 2022-04-19

**Authors:** Momal Sharif, Vincent Hickl, Gabriel Juarez, Xingjian Di, Karl Kerns, Peter Sutovsky, Nicolai Bovin, David J. Miller

**Affiliations:** 1 Department of Animal Sciences and Institute of Genomic Biology, University of Illinois at Urbana-Champaign, 1207 West Gregory Drive, Urbana, IL 61801 USA; 2 Department of Physics, University of Illinois at Urbana-Champaign, Urbana, IL 61801 USA; 3 Department of Mechanical Science and Engineering, University of Illinois at Urbana-Champaign, Urbana, IL 61801 USA; 4 Department of Animal Science, Iowa State University, Ames, IA 50011 USA; 5 Division of Animal Sciences, University of Missouri, Columbia, MO 65211 USA; 6 Department of Obstetrics, Gynecology and Women’s Health, University of Missouri, Columbia, MO 65211 USA; 7 Shemyakin Institute of Bioorganic Chemistry, Moscow, Russia; 8 Present Address: Department of Obstetrics and Gynecology, Baylor College of Medicine and Jan and Dan Duncan Neurological Research Institute, Texas Children’s Hospital, Houston, TX 77030 USA

**Keywords:** Germ cells, Glycobiology

## Abstract

Fertilizing sperm are retained by adhesion to specific glycans on the epithelium of the oviduct forming a reservoir before sperm are released from the reservoir so fertilization can ensue. Capacitated sperm lose affinity for the oviduct epithelium but the components of capacitation that are important for sperm release are uncertain. One important correlate of capacitation is the development of hyperactivated motility. Hyperactivation is characterized by asymmetrical flagellar beating with high beat amplitude. We tested whether the development of full-type asymmetrical motility was sufficient to release sperm from immobilized oviduct glycans. Sperm hyperactivation was induced by four different compounds, a cell-permeable cAMP analog (cBiMPS), CatSper activators (4-aminopyridine and procaine), and an endogenous steroid (progesterone). Using standard analysis (CASA) and direct visualization with high-speed video microscopy, we first confirmed that all four compounds induced hyperactivation. Subsequently, sperm were allowed to bind to immobilized oviduct glycans, and compounds or vehicle controls were added. All compounds caused sperm release from immobilized glycans, demonstrating that hyperactivation was sufficient to release sperm from oviduct cells and immobilized glycans. Pharmacological inhibition of the non-genomic progesterone receptor and CatSper diminished sperm release from oviduct glycans. Inhibition of the proteolytic activities of the ubiquitin–proteasome system (UPS), implicated in the regulation of sperm capacitation, diminished sperm release in response to all hyperactivation inducers. In summary, induction of sperm hyperactivation was sufficient to induce sperm release from immobilized oviduct glycans and release was dependent on CatSper and the UPS.

## Introduction

In many animals, sperm are stored after semen deposition for a period of time before fertilization ^1^ Sperm storage time ranges from a day to years, depending on the species ^2^. In mammals, the lower oviduct, known as the isthmus, is the major site of sperm storage before fertilization ^1^. Species-dependent glycans on the surface of the oviduct epithelium bind and retain sperm in the isthmus ^3^. Recent studies using porcine cells have demonstrated that sperm bind specifically to glycans containing either of two specific motifs, 3-O-sulfated Lewis X trisaccharide (suLe^X^) or a branched oligosaccharide with 6-sialylated lactosamine termini (bi-SiaLN), which are abundant on the isthmic epithelium ^4–7^. Binding to these glycans suppresses the normal increase in intracellular Ca^2+^ in sperm and extends the lifespan of sperm in vitro ^8,9^.

Sperm stored in the isthmus must be released from the epithelium to move to the ampulla (upper oviduct) to fertilize oocytes. There are many reports that, once sperm have completed the final maturation process to give them fertilizing ability, known as capacitation, they are no longer able to bind to the oviduct epithelium and are thereby found in the lumen of the oviduct ^5,6,10–12^. This led to the hypothesis that capacitation is the activator of sperm release from the isthmus. But capacitation includes a variety of processes such as changes in the sperm membrane including lipid alterations, protein migration, or degradation, including possible degradation of glycan receptors during capacitation ^9,13^. Finally, as sperm capacitate, they develop an altered tail beat pattern known as hyperactivation ^14–17^. Although the specific patterns of hyperactivated motility differ some between species, hyperactivated sperm have two distinct types of movements, described recently in porcine sperm ^15,18,19^. Initially, “asymmetrical” motility develops that is characterized by bending of the middle and principal pieces of sperm more towards one side than the other in aqueous media and more progressive movement in viscous media. In sperm with greater hyperactivation, an “extremely asymmetrical” pattern is characterized by further exaggerated bending of the middle and principal pieces and by figure eight-like movement or twisting movement in an aqueous medium. This movement has also been termed full-type hyperactivation ^15,18,19^. Hyperactivation may provide the force to pull sperm away from their oviduct epithelial attachment sites ^20^.

Development of hyperactivated motility requires the function of the major Ca^2+^ channels in sperm, known as CatSper channels ^21–26^. Although CatSper has not been well-studied in porcine sperm, mouse or human sperm that lack CatSper subunits are infertile ^25,27^ and sperm deficient in CatSper are unable to hyperactivate or pass through the utero-tubal junction (UTJ) to enter the oviduct ^1,25,27–32^. CatSper channels are only found in sperm and are activated by changes in internal pH or by progesterone in mouse or human sperm, respectively ^21,33–38^. Progesterone stimulates human sperm hyperactivation by binding to a non-genomic receptor on the plasma membrane, abhydrolase domain-containing protein 2 (ABHD2), modulating the endocannabinoid system, and opening CatSper channels ^39^. The importance of CatSper emphasizes the central role that Ca^2+^ plays in sperm hyperactivation ^40^. Other signaling systems are also engaged to stimulate sperm hyperactivation including those activated by the second messenger cAMP ^41–44^.

Because capacitation and hyperactivation are usually coupled, it is not clear whether the release is due to capacitation and its associated membrane modifications ^45,46^ or specifically to hyperactivation ^20,47,48^. To distinguish if hyperactivation and not the other capacitation-associated changes can trigger sperm release, one can add pharmacological agents to uncapacitated sperm that affect intracellular signaling systems and engage hyperactivated motility before the normal time required for sperm to complete capacitation. There are several agents reported to trigger sperm hyperactivation. For example, procaine^49^, 4-aminopyridine (which both activate CatSper indirectly)^49^, and the cell-permeable cAMP analog Sp-5,6-dichloro-1-b-D-ribofuranosyl-benzimidazole-30,50-monophosphorothioate induce full-type hyperactivation of porcine sperm ^18,50^, which otherwise seldom undergo significant full-type hyperactivation in a capacitating medium, unlike mouse epididymal sperm^19,50,51^. Following the addition of these pharmacological agents, porcine sperm display a relatively high percentage of full-type hyperactivated motility before the time that sperm have completed capacitation^18,50^. This approach allows the separation of hyperactivation from other events in capacitation, which require 5–6 h ^52^.

The purpose of this study was to determine if sperm hyperactivation was sufficient to promote sperm release from oviduct cells and immobilized glycans. Additionally, we investigated the role of sperm protein degradation by the ubiquitin proteasomal system (UPS) during sperm hyperactivation. We have recently described how progesterone-induced sperm release from the oviduct reservoir requires both active CatSper channels and the function of the ubiquitin proteasomal system ^13^. Earlier studies provided compelling evidence of UPS being a regulating element of sperm capacitation and fertilization ^53–55^ but the possible interaction between hyperactivation and the UPS has not been explored. The UPS functions broadly in protein degradation including degradation of sperm proteins such as AKAP3^56^ and the release of decapacitating factors such as spermadhesins that bind spermatozoa to oviductal epithelia^13,54^. Herein we also examine whether the UPS is necessary for sperm release when uncapacitated sperm are stimulated to hyperactivate.

## Materials and methods

### Collection and processing of sperm

For each replicate, semen was sourced from Prairie State Semen Supply, Champaign, IL or PIC, Hendersonville, TN, collected from at least 3 mature boars by ejaculation induced by manual pressure on the glans penis. Semen was extended in Preserve XLT extender (Continental Plastics, WI) to 3 billion cells per 80 ml dose, cooled to 17 °C, transported to the laboratory, and processed within 24 h, as described before ^8,9,13^.The extended semen was pooled and 3 mL were washed through a Percoll cushion containing a mixture of 4 mL of dmTALP (2.1 mM CaCl_2_, 3.1 mM KCl, 1.5 mM MgCl_2_, 100 mM NaCl, 0.29 mM KH_2_PO_4_, 0.36% lactic acid, 26 mM NaHCO_3_, 0.6% BSA, 1 mM pyruvic acid, 20 mM HEPES pH 7.3, sterile filtered), 0.6 mL of 10X HBS (1.3 M NaCl, 40 mM KCL, 10 mM CaCl_2_, 5 mM MgCl_2_, 140 mM fructose, 5% BSA, sterile filtered), and 5.4 mL of Percoll for 10 min at 800 × g. The supernatant was discarded, and the resulting pellet was re-suspended in 14 mL of dmTALP and centrifuged for 3 min at 600 × g. Once again, the supernatant was discarded, and the resulting pellet was re-suspended in 1 mL of dmTALP. Sperm concentration was estimated by hemocytometer and only samples with greater than 75–80% motile sperm were used for experiments.

### Effects of hyperactivation-inducing compounds on sperm motility

The sperm were incubated with cBiMPS (50 and 100 μM; from stock of 4.771 mM), 4-aminopyridine (4-AP; 2 and 4 mM; from stock of 500 mM), procaine (2.5 and 5 mM; from stock of 100 mM), progesterone (80 nM; from stock of 1 μM), or vehicle control. All pharmacological compounds were dissolved in 10% (v/v) dimethyl sulfoxide (DMSO) to generate stock solutions that were then further diluted in dmTALP depending on treatment/compound concentration. The same volume of 10% DMSO was diluted in dmTALP and added to equalize the concentration of solvent for the vehicle control. The motility was assessed using a Hamilton Thorne Semen Analysis CASA System (Hamilton Thorne, Beverly, MA, USA). The initial concentrations of these compounds were selected based on previous reports (Supplementary Table S1) ^18,19^. Sperm were incubated with each compound at 39 °C in dmTALP for 30 min. For each experimental condition, 5 random fields were evaluated for a minimum total of 100 cells (in each field) in 5 replicates.

Direct visualization with high-speed video microscopy was used to evaluate sperm motility on the treatments showing significant changes in CASA evaluation, cBiMPS (100 μM), 4-aminopyridine (4-AP, 4 mM), procaine (5 mM), progesterone (80 nM), and vehicle control. After 30 min of incubation as described, 20 μl of sperm suspension was placed on a slide and a coverslip was added. The volume of sperm suspension was purposely limited to immobilize sperm and assess the motility pattern. Imaging was done using a motorized inverted microscope (Nikon Eclipse Ti). All images were taken using phase-contrast microscopy at 200X magnification. High-speed videos of 10 s each were recorded at 100 fps with a 10 ms exposure time.

Every tenth image (100 ms apart) was collected to create a minimum-intensity projection image (MinIP) using Nikon’s NIS-Elements software. The algorithm uses all the data in a video to generate a single 2-dimensional image. Each sperm in the image had localized pixels (darker values) in 100 images over 10 s. The resulting images were quantified in two ways. First, sperm were categorized as either static or motile. Motile sperm were placed in one of three groups (progressive, asymmetrical, and symmetrical). Second, the asymmetry of motility was estimated in sperm in which the heads were attached to the slide. Attached sperm with asymmetric tail beat patterns pivoted at the point where the head was attached. More asymmetrical patterns rotated further around the pivot point, as much as 360°. Sperm with less asymmetrical beat patterns rotated the head to a lesser degree. Sperm were assigned into one of six groups based on the degree of rotation around the head (0°), (1–90°), (91–180°), (181–359°), (360°) or not applicable (N/A). The N/A category included highly clumped (self-agglutinated) sperm with an indistinguishable degree of rotation.

### Assay of sperm release from glycan-coupled beads

Glycan-coated streptavidin-Sepharose High-Performance Beads (GE Healthcare Bio-Sciences, Pittsburgh, PA) with an average diameter of 34 µm were used to test the ability of porcine sperm binding to glycans (bi-SiaLN and suLe^X^), as described previously ^13^. To conjugate glycans to beads, approximately 60 µg of suLe^X^ and 60 µg of bi-SiaLN ^57^ were covalently attached to a biotinylated polyacrylamide core were, together, incubated with 20 µL of streptavidin-Sepharose beads for 90 min at room temperature . Each 20-kDa molecule of polyacrylamide had 20% glycan and 5% biotin, by molarity. Beads incubated with glycans were washed twice in dmTALP and re-suspended in 100 µL of dmTALP. Once the glycan-coupled beads were ready for use, a 50 µL-droplet containing 1.5 × 10^6^ sperm/mL was prepared to receive 1 µL of glycan-coated beads (Supplementary Figure S1). Sperm and beads were co-incubated at 45 min at 39 °C. After that, either cBiMPS (100 μM), 4-AP (4 mM), procaine (5 mM), progesterone (80 nM), or vehicle control were added. After incubation for 30 min at 39 °C_,_ for each treatment, 25 beads were randomly selected, and the total number of bound sperm was enumerated in three replicates droplets using a Zeiss Axioskop at 200X magnification. Sperm that were self-agglutinated were not included in the counts. At least three biological replicates each with three triplicate droplets were done for each treatment. The experiments were documented using an Axiocam and AxioVision or ZEN software (Zeiss, Thornwood, NY).

### Effect of proteasomal degradation, CatSper and progesterone receptor inhibitors on sperm release

To test the role of CatSper and progesterone, sperm were allowed to bind to glycan-coated beads as stated earlier. A proteasomal inhibitor cocktail including MG132, clasto-lactacystin μ-lactone (CLBL), and epoxomicin (10 μM of each inhibitor from the stock of 1 mM), referred to as MCE (Supplementary Table S1), ^13^ was added to the sperm bound to glycan coated beads 15 min before addition of the hyperactivation compounds cBiMPS (100 μM), 4-AP (4 mM), procaine (5 mM), progesterone (80 nM) or vehicle control. Alternately 2 µM of a T channel blocker (NNC 055–0396 from a 10 mM stock) or 2 µM of a blocker of the non-genomic progesterone receptor, methoxy arachidonyl fluorophosphonate (MAFP from a 10 mM stock), as described in ^9^, was added to the sperm bound to glycan coated beads 15 min before addition of hyperactivation compounds. Sperm were incubated with the hyperactivation compounds for 30 min before imaging. For each treatment, 25 beads were randomly selected, and the total number of bound sperm was enumerated in three replicate droplets. At least three biological replicates that included three droplets were performed for each treatment. The experiment was documented using a Zeiss Axioskop at 200X magnification (Zeiss, Thornwood, NY).

### Statistical analysis

Each sperm binding experiment was performed at least 3 times using triplicate droplets. Differences among means were determined using a one-way analysis of variance in SAS (v. 9.1 SAS Institute, Inc, Cary, NC). A droplet containing sperm bound to beads was considered an experimental unit. Percent inhibition of sperm release was calculated by dividing the difference between “before treatment” and “after pharmacological inhibitor is added” by the control treatment without any inhibitor. The results are shown as means ± SEM and the means were considered to belong to distinct populations if *P* < 0.05 using Tukey’s test for multiple comparisons.

## Results

### Effects of hyperactivity inducers on sperm motility

First, we confirmed the ability of progesterone and other activators of CatSper as well as a cell-permeable analog of cAMP to induce hyperactivated motility. Sperm were incubated for 30 min with cBiMPS (50 and 100 μM), 4-aminopyridine (4-AP, 2 and 4 mM), procaine (2.5 and 5 mM), progesterone (80 nM), or vehicle control. Motility was first evaluated by CASA, which assesses tail motion indirectly by measuring the movement of the sperm head. There were significant differences in sperm motility characteristics within 30 min. Only the results from higher concentrations are shown (Table 1).

**Table 1:** Effects of hyperactivity-inducing compounds on sperm motility measured by CASA.

Treatments	P4	4-AP	cBIMPS	Procaine	VC
Progressive %	35.0 ± 2.2	40.8 ± 4.0	40.6 ± 4.5	39.4 ± 5.8	26.6 ± 2.7
Motility %	61.6 ± 3.6	70.2 ± 4.8	69.6 ± 3.7	71.8 ± 3.7	67.8 ± 2.3
Rapid %	36.2 ± 8.1	55.2 ± 3.8	51.0 ± 5.0	63.0 ± 6.4	28.4 ± 2.0
VAP μm/sec	70.22 ± 8.9	80.26 ± 6.1	82.3 ± 8.2	98.78 ± 14.8	49.2 ± 6.0
VSL μm/sec	46.6 ± 7.0	26.6 ± 6.1	50.26 ± 3.8	43.04 ± 5.3	30.66 ± 2.2
VCL μm/sec	151.44 ± 9.8	180.84 ± 28.8	156.26 ± 33.8	228.76 ± 43.6	110.28 ± 13.3
ALH μm	8.58 ± 0.9*	11.28 ± 0.9*	8.32 ± 1.6*	9.76 ± 0.8*	7.44 ± 1.6
BCF Hz	39.24 ± 1.7*	48.62 ± 6.3*	39.48 ± 6.7*	40.34 ± 4.1*	37.98 ± 2.0
Straightness %	60.4 ± 5.7	41.0 ± 19.1	59.8 ± 4.7	43.8 ± 3.2	62.0 ± 9.7
Linearity %	29.4 ± 5.0	20.4 ± 4.6	36.0 ± 3.8	19.6 ± 3.6	29.4 ± 4.6
Elongation %	38.4 ± 2.0	44.4 ± 5.7	42.6 ± 4.8	46.4 ± 5.9	39.6 ± 4.7
Area μm^2^	18.38 ± 2.6	9.44 ± 1.9	14.56 ± 2.1	13.74 ± 4.6	10.14 ± 2.3

Motility parameters of sperm incubated with cBiMPS (100 μM), 4-AP (4 mM), procaine (5 mM), progesterone (80 nM), and vehicle control 30 min after addition. Results are means and standard deviations.

VAP = average path velocity; VSL = straight-line velocity; VCL = curvilinear velocity; ALH = amplitude of lateral head displacement; BCF = beat cross frequency.

Asterisks represent significant differences from vehicle control for ALH and BCF. n = 5 replicates.

The most potent of the four tested compounds was 4-AP, but all compounds induced an increase in the amplitude of lateral head displacement (ALH). That is, with each tail beat stroke, the sperm head deviated to a greater degree from a line representing the net movement of the head. Each compound also induced an increase in tail beat frequency (BCF). Again, 4-AP induced the greatest increase. These changes are consistent with the induction of asymmetrical full-type hyperactivated motility by porcine sperm.

**Figure 1:**
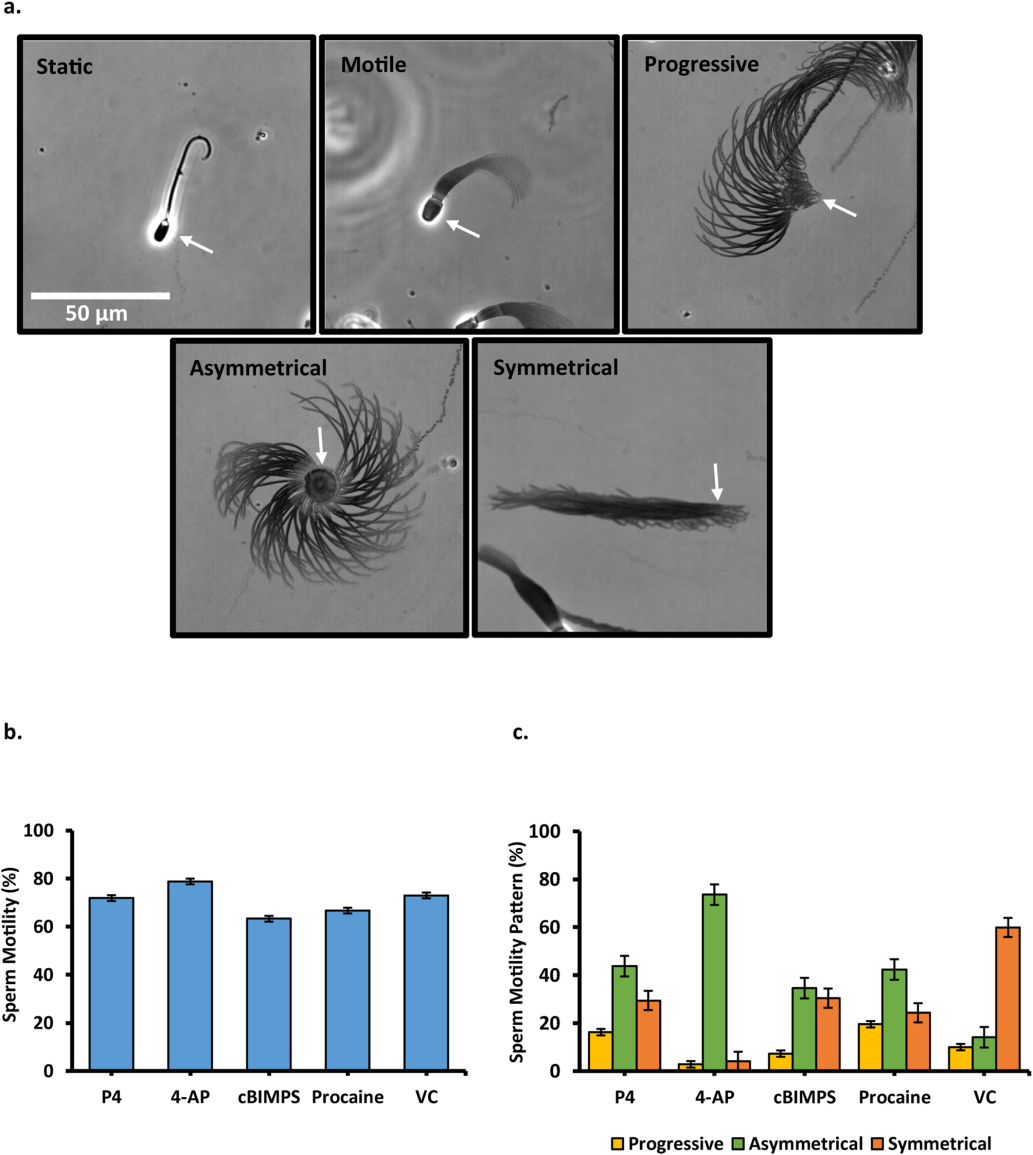
Effects of hyperactivity inducers on sperm motility patterns (**a**) Examples of sperm that displayed specific motility patterns observed when single images compiled from high-speed video microscopy were used to categorize sperm into five groups (static, motile, progressive, asymmetrical, and symmetrical). The white arrows label the sperm heads. Immotile sperm were placed in the “static” category and all sperm showing movement were placed in the “motile” category. The motile sperm were placed into one of either “progressive”, “asymmetrical”, or “symmetrical” categories. The scale bar indicates 50 μm in length (**b**) Graph of the percentage of the motile sperm in P4, progesterone; 4-AP, 4-aminopyridine; cBIMPS; Procaine; VC, vehicle control. n = 4 replicates, with 350–500 sperm per replicate (**c**) Graph of the percentage of the total sperm within each motility group. The same sperm could be placed into more than one group (i.e., progressive, and asymmetrical or symmetrical). Thus, the totals do not equal 100. P4, progesterone; 4-AP, 4-aminopyridine; cBIMPS; Procaine; VC, vehicle control. n = 4 replicates, with 350–600 sperm per replicate. All treatments were significantly different from each other and vehicle controls.

The motility patterns of sperm exposed to cBiMPS (100 μM), 4-AP (4 mM), procaine (5 mM), progesterone (80 nM), and vehicle control were also assessed using high-speed video microscopy as described in Material and Methods. From each video, a single minimum-intensity projection image (MinIP) was generated which was used to evaluate tail motion. First, sperm were categorized as either static or motile. Then motile sperm were described, based on evaluation of the image generated from the movie, as having motility that was either progressive, asymmetrical, or symmetrical. An example of each is shown (Fig. 1a). The groups were not mutually exclusive; that is, the same sperm could be placed into more than one group. For example, a single sperm could be motile, and its motility could be symmetrical. Sperm motility percentage in each treatment was not significantly different in any treatment compared to the control (Fig. 1b). All hyperactivity inducers increased the percentage of sperm with asymmetrical motility (Fig. 1c). There were significant differences in sperm motility patterns in all treatments compared to the vehicle control (Fig. 1c). The greatest percentage of sperm showing asymmetric motility was seen in 4-AP treated cells and the vehicle control (VC) yielded the highest percentage of sperm with symmetrical motility.

**Figure 2:**
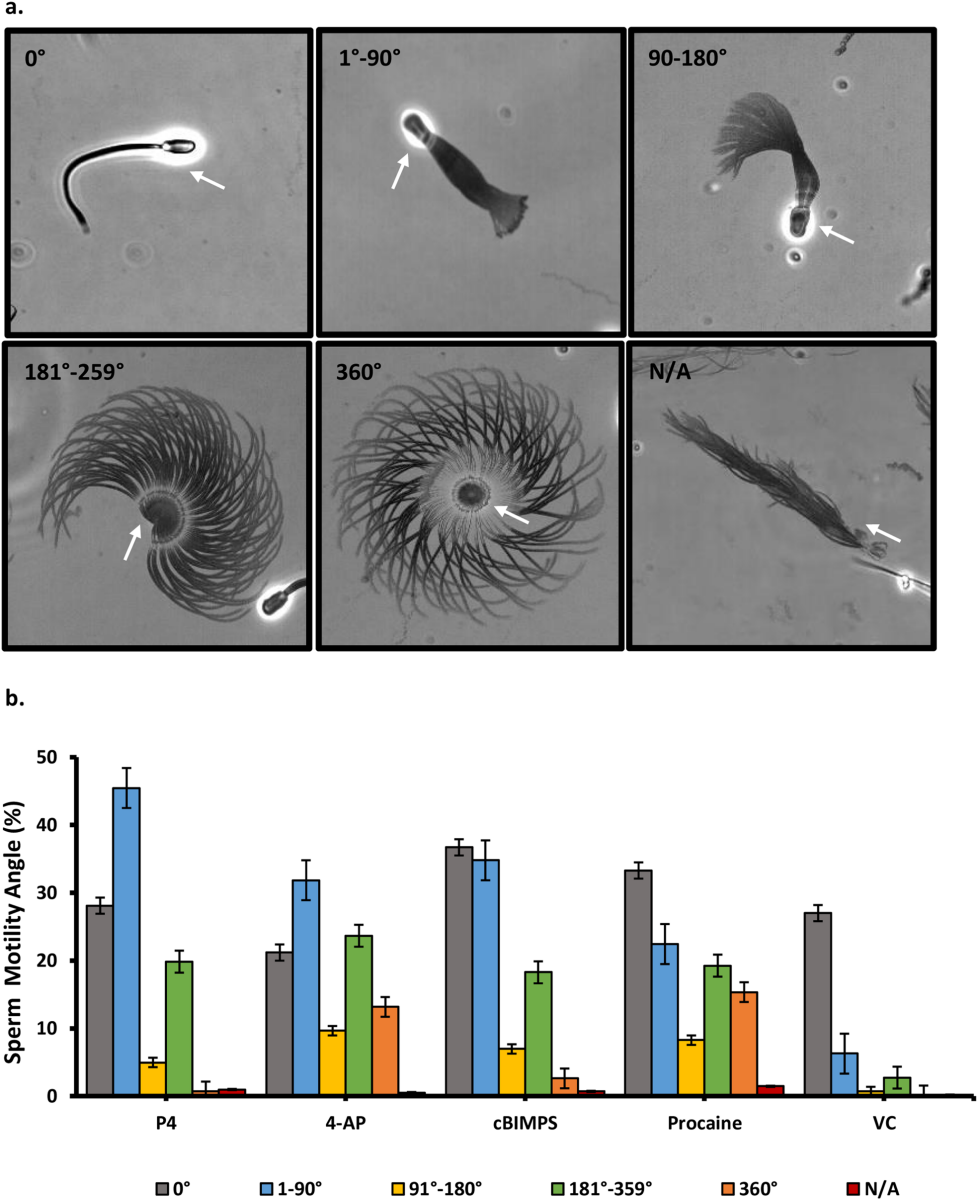
Effects of hyperactivation inducers on sperm asymmetry (**a**) Representative images of sperm obtained from high-speed video used to assign into six groups of geometric angles representing rotations of sperm (0°), (1–90°), (91–180°), (181–359°), (360°), and not applicable (N/A) (**b**) Percentage of total sperm observed that were within each range of rotation. White arrows point towards the sperm head. n = 4 replicates with 350–500 sperm per replicate. All treatments were significantly different from each other and vehicle controls.

The heads of many sperm adhered to the microscope slide and if their tail was beating asymmetrically, the sperm would rotate around the adherent head. This was observed easily in MinIP (Fig. 2a). The sperm rotation was categorized in 90° increments. Procaine and 4-AP yielded the highest full-type hyperactivation because samples with these compounds had the highest number of cells showing 360° and 180–360° angles (Fig. 2b). Thus, by both assessment of sperm head motion using CASA and independent assessment of tail motion using high-speed video microscopy, the compounds tested induced hyperactivation of porcine sperm.

### Hyperactivation is sufficient for sperm release from immobilized oviduct glycans

**Figure 3:**
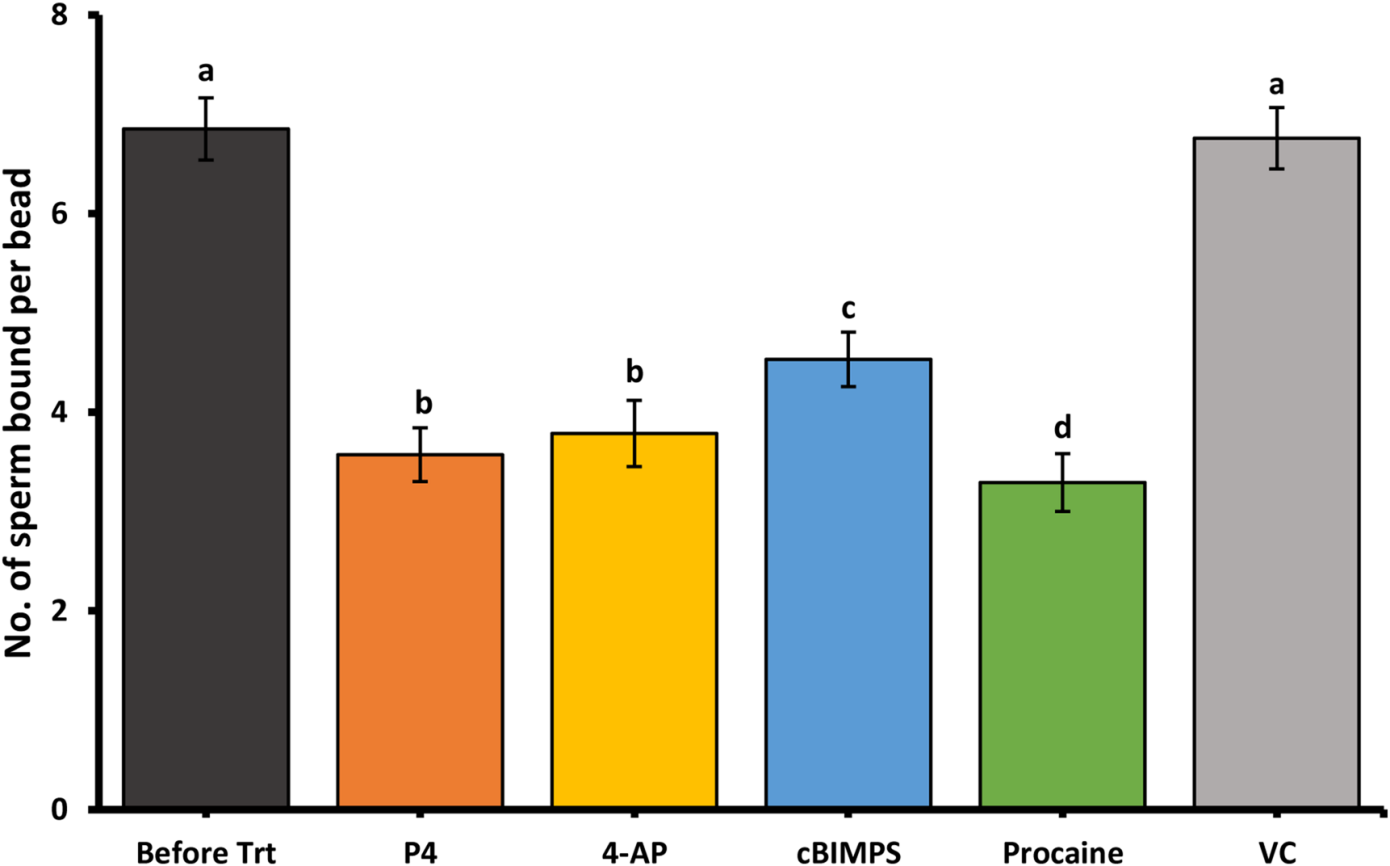
Hyperactivation is sufficient for sperm release from oviductal glycans. The number of sperm bound per glycan-coated bead coated in capacitating medium, before and following a 30 min incubation with cBiMPS (100 μM), 4-AP (4 mM), procaine (5 mM) or progesterone (80 nM). All hyperactivity inducers promoted sperm release from immobilized oviduct glycans when compared with controls. Groups that are significantly different are labeled with different letters (*P* < 0.05), n = 3 replicates.

We used an in vitro assay to test the ability of hyperactivating compounds to release porcine sperm from oviduct glycans attached to beads. Both suLe^X^ and bi-SiaLN together were attached to the same beads because both bind sperm and are found on oviduct epithelial cells ^5,58^. The ability of cBiMPS (100 μM), 4-AP (4 mM), procaine (5 mM), and progesterone (80 nM) to release sperm from oviduct glycan-coated beads was determined. All four compounds induced sperm release from immobilized oviduct glycans. The number of sperm bound to immobilized glycans was reduced by the four hyperactivation inducers compared to the number of bound sperm before their addition or following the addition of vehicle control (Fig. 3). Each released a number of sperm that was similar to the number released by progesterone. Therefore, hyperactivity inducers cBiMPS, 4-AP and procaine were individually sufficient for sperm release from immobilized oviduct glycans.

### Inhibitors of the non-genomic progesterone receptor and CatSper block sperm release due to hyperactivation

**Figure 4:**
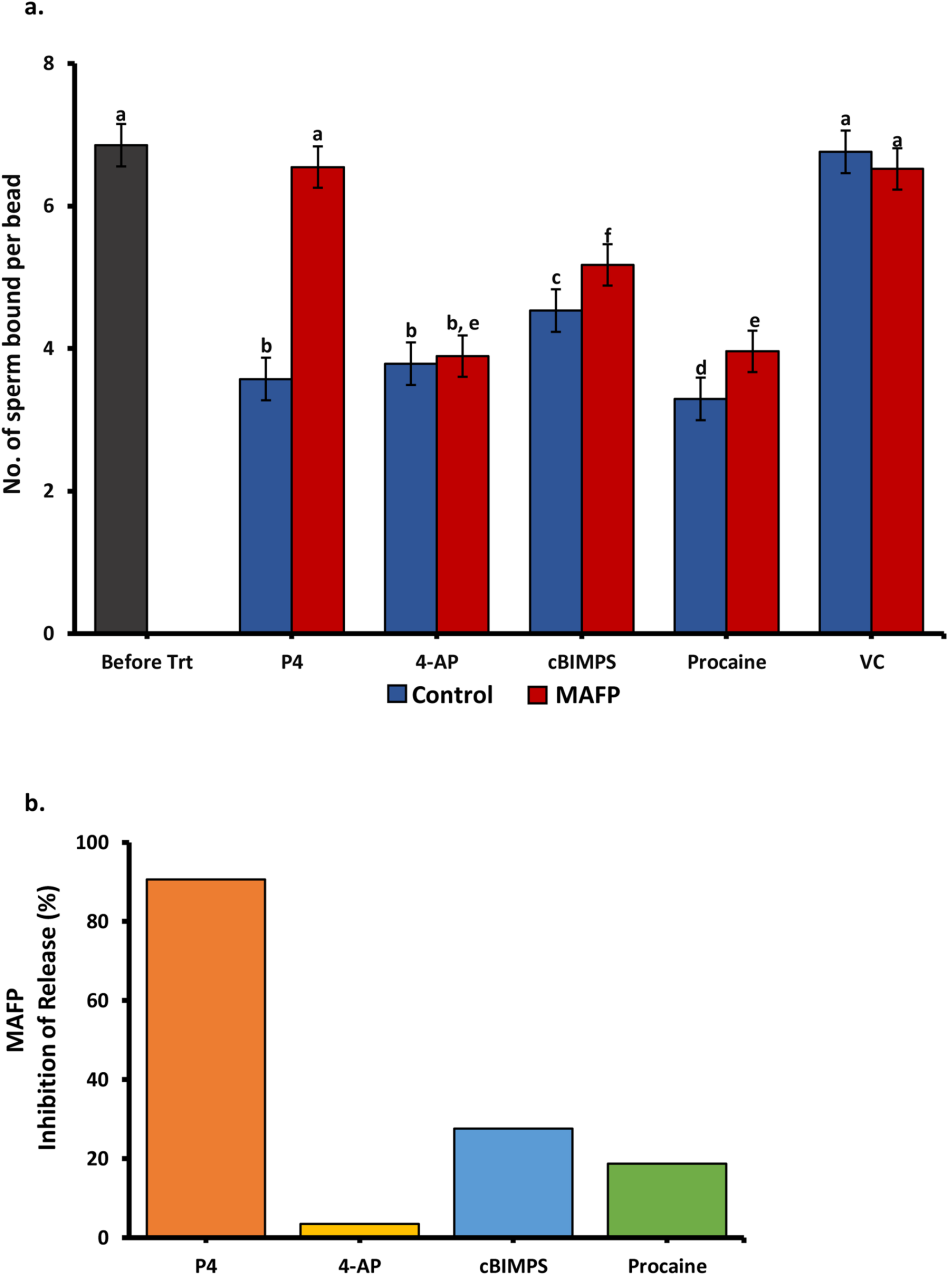
Inhibition of ABHD2 modulates sperm release in response to progesterone (**a**) The number of sperm bound per oviduct glycan-coated bead (suLe^X^ and bi-SiaLN combined on beads) before and after addition of hyperactivation inducing compounds, with and without the non-genomic progesterone receptor inhibitor methoxy arachidonyl fluorophosphonate (MAFP). To induce sperm release from immobilized oviduct glycans, cBiMPS (100 μM), 4-AP (4 mM), procaine (5 mM), progesterone (80 nM), or vehicle control (VC) were tested. Groups that are significantly different are labeled with different letters (*P* < 0.05), n = 3 replicates.; n = 3 replicates (**b**) The percentage of sperm release was inhibited by MAFP. Progesterone-induced sperm release was completely blocked by MAFP. Sperm release in response to cBiMPS, 4-AP, and procaine was only partially inhibited or unaffected by MAFP.

In human sperm, progesterone binds to a membrane receptor, abhydrolase domain-containing protein 2 (ABHD2), a serine hydrolase^39,59^. ABHD2 in human sperm is inhibited by methoxy arachidonyl fluorophosphonate (MAFP), a serine hydrolase inhibitor^39,59^. To determine if progesterone acted on porcine sperm by binding ABHD2, MAFP was added to sperm bound to beads before the addition of progesterone. MAFP completely blocked sperm release induced by progesterone (Fig. 4a). Next, we investigated if blocking ABHD2 inhibited sperm release from compounds that activated signaling that was downstream of ABHD2. MAFP did not affect sperm release stimulated by 4-AP and had only a minimal effect on sperm release induced by cBiMPS and procaine (Fig. 4b). Therefore, in porcine sperm, progesterone stimulated sperm hyperactivation through ABHD2 but 4-AP, cBiMPS, and procaine mostly bypassed ABHD2 to stimulate hyperactivation.

**Figure 5:**
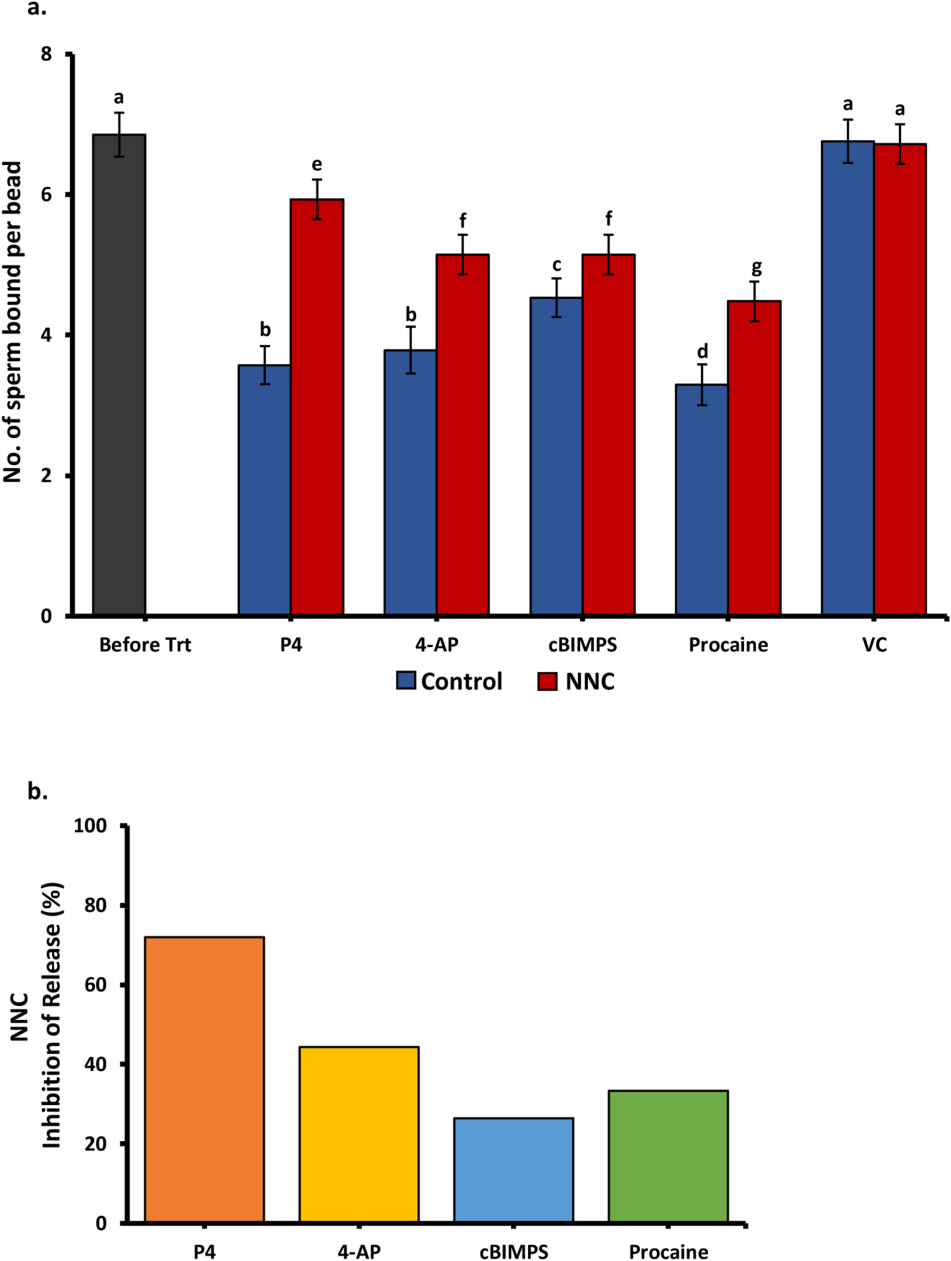
Inhibition of CatSper by NNC 055–0396 (NNC) affected sperm release in response to hyperactivation inducers (**a**) The number of sperm bound per oviduct glycan-coated bead before and after addition of hyperactivation-inducing compounds with and without NNC. cBiMPS (100 μM), 4-AP (4 mM), procaine (5 mM), progesterone (80 nM), or vehicle control (VC) were used to induce sperm release from immobilized oviduct glycans (**b**) The percentage of sperm release that was inhibited by NNC. Sperm release induced by progesterone was almost completely blocked by NNC. NNC had less inhibition of sperm release in response to cBiMPS, 4-AP, and procaine. Groups that are significantly different are labeled with different letters (*P* < 0.05), n = 3 replicates.; n = 3 replicates for each panel.

In human sperm, once progesterone binds to ABHD2, ABHD2 cleaves membrane 2 arachidonoylglycerol, releasing its tonic inhibition of CatSper, allowing Ca^2+^ influx into sperm ^39^. The T channel inhibitor NNC 055–0396 at a concentration that abolishes CatSper currents in human sperm but does not have untoward effects (2 µM) ^36,60^, was examined to determine if it would diminish the induced sperm release. The NNC compound blocked release induced by progesterone (Fig. 5a). Interestingly, blocking CatSper partially blocked sperm release induced by the activator of the cAMP pathway, cBIMPS as well as the CatSper activators 4-AP and procaine (Fig. 5b).

### Inhibitors of the 20S proteasomal core activities block sperm release due to hyperactivation

**Figure 6:**
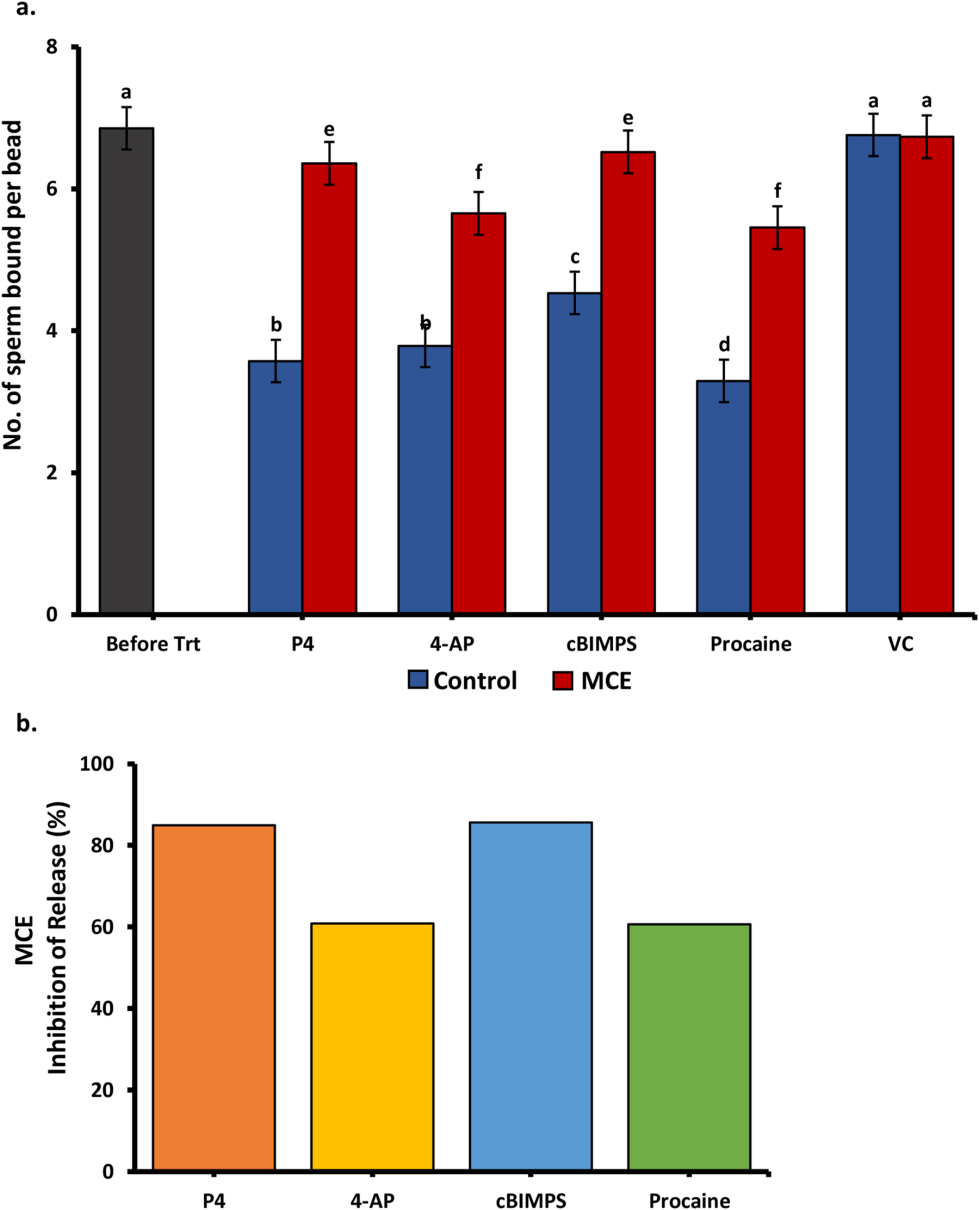
Proteasomal inhibitors affected sperm release in response to hyperactivation inducers (**a**) The number of sperm bound per oviduct glycan-coated bead before and after addition of hyperactivation inducing compounds with and without proteasomal inhibitor cocktail MCE (10 μM). cBiMPS (100 μM), 4-AP (4 mM), procaine (5 mM), progesterone (80 nM), or vehicle control (VC) were used to induce sperm release from immobilized oviduct glycans (**b**) The percentage of sperm release that was inhibited by the proteasomal inhibitor cocktail (MCE). The proteasomal inhibitors diminished sperm release induced by progesterone, cBIMPS, 4-AP, and procaine. Groups that are significantly different are labeled with different letters (*P* < 0.05), n = 3 replicates.; n = 3 replicates for each panel.

Because of the potential role of the ubiquitin-proteasomal system in sperm release induced by progesterone ^13,54,61,62^, we investigated the possibility that protein degradation by the UPS was involved broadly in induced sperm release. We preincubated sperm bound to immobilized glycans in a cocktail of 3 proteasomal inhibitors (MCE), MG132, clasto-lactacystin β-lactone (CLBL), and epoxomicin. We added either 4-AP, cBIMPS, or procaine as well as progesterone as a positive control. The proteasomal inhibitor cocktail, which specifically inhibits proteolytic activities within the 20S proteasomal core without affecting conventional cellular proteases, nearly eliminated sperm release induced by progesterone (Fig. 6a). It also diminished the release of most sperm following exposure to all three pharmacological hyperactivity inducers (Fig. 6b). These results suggest that the UPS is necessary for sperm release and that the signaling pathways that provoke hyperactivation also promote proteasomal degradation of sperm proteins involved in binding to the oviduct.

## Discussion

The sperm reservoir in the isthmus of mammals supplies sperm to the ampulla where they can fertilize oocytes. Porcine sperm are retained in the isthmus by adherence to glycans on the epithelium that contain either of two specific structural motifs. Although sperm must be released from the reservoir for fertilization, an understanding of how sperm are released has been elusive. It is clear that capacitated sperm have lost their affinity for the oviduct epithelium ^5,6,63^ but the component/s of the complex process of capacitation that are responsible for the release of sperm are indefinite. One of many notable changes that sperm undergo during capacitation is the development of a hyperactivated form of motility, characterized by high-amplitude asymmetrical flagellar bending ^51^. Hyperactivated motility has been proposed to enable sperm to escape from the oviduct isthmic reservoir because CatSper -/- sperm that do not hyperactivate cannot ascend beyond the isthmus to fertilize oocytes ^27,64^. Whether the function of hyperactivation is for sperm release from epithelial glycans, for sperm movement in the viscous fluid of the oviduct lumen, or something else is an important subject to clarify. As hyperactivation is linked to sperm capacitation, it has been challenging to distinguish which processes of capacitation are important for sperm release. The discovery that several pharmacological agents could induce sperm hyperactivation in uncapacitated sperm allowed the evaluation of the effects of hyperactivation separately from other events that happen during sperm capacitation, such as membrane modifications ^18,50,51^. Herein, we used compounds that can induce hyperactivation in sperm that are not capacitated, thus allowing the dissociation of capacitation and hyperactivation ^51^.

In the present study, we characterized the ability of four compounds, an endogenous steroid (progesterone) and three different types of pharmacological agents (cBiMPS, 4-AP, and procaine), that act on different signaling steps that converge to open CatSper channels and stimulate full-type hyperactivated motility to release sperm from two immobilized oviduct glycans. These glycans act as sperm receptors on the apical surface of the oviductal epithelium. Results showed that the tested compounds induced hyperactivated sperm motility before the time capacitation would be completed. Of the four inducers, at the concentrations tested, 4-AP and procaine were the most potent inducers of hyperactivated motility. All four compounds also induced sperm release from oviduct glycans within 30 min. The fact that these compounds induce hyperactivation by activating different intracellular signaling pathways, CatSper/Ca^2+^, and cAMP, but still induce sperm release, suggests that the mechanism by which they induce release specifically promotes hyperactivated motility rather than by some other phenomenon or off-target effect.

Inhibitors of the non-genomic progesterone receptor ABHD2 (MAFP), identified in human sperm, of CatSper (NNC 055-0396) and of the UPS (MG132, clasto-lactacystin β-lactone, and epoxomicin) were tested for their effects on sperm release. As expected, MAFP, the ABHD2 inhibitor ^39,59^ blocked release by progesterone but had little effect on release in response to any of the hyperactivity inducers. This was expected because the other compounds that induce hyperactivation act downstream of ABHD2.

Because CatSper is necessary for hyperactivation, we tested whether an inhibitor of CatSper, NNC 055–0396, affected sperm release. The NNC compound minimally reduced sperm release due to activation of the cAMP pathway, suggesting that CatSper is not downstream of cAMP/Protein Kinase A activation. This is consistent with a recent report ^65^ but in contrast to previous studies that concluded CatSper was activated by the cAMP/Protein Kinase A pathway ^24,66^. Furthermore, blocking CatSper partially inhibited sperm release activated by 4-AP and procaine. Although 4-AP is said to be an activator of CatSper ^40^ indirectly by activating Kv channels ^67^, the complete actions of 4-AP on sperm are uncertain. It is known that millimolar concentrations of 4-AP induce hyperactivation in sperm deficient in CatSper^68^ and can activate other Ca^2+^ channels directly ^69^. Procaine appears to activate CatSper indirectly ^29^. There is evidence that procaine promotes partial hyperactivation of mouse sperm deficient in CatSper ^25^. This indicates that there are targets of both 4-AP and procaine in sperm in addition to CatSper that can affect hyperactivation. This may explain why NNC only partially inhibits sperm release in response to 4-AP and procaine.

Previous studies have shown that the UPS was necessary for sperm release ^13^. The activation of the UPS may be through cAMP/PKA-induced phosphorylation of a proteasomal subunit that is also induced during capacitation ^70^. Our results that inhibition of protein degradation by proteasomes diminished sperm release triggered by each hyperactivation inducer further support this hypothesis. Interestingly, porcine sperm candidates for oviduct glycan receptors, adsorbed onto sperm surface from seminal plasma, were co-precipitated with proteasomal subunits ^71^, suggesting that their degradation or degradation of cofactors that hold them on the sperm surface might be involved with sperm release. This indicates that the signaling systems that induce sperm hyperactivation appear to also evoke proteolysis of some membrane proteins including an unknown number that may be important for sperm adhesion to the oviduct. An alternative target of proteasomes may be tail proteins, such as AKAP3, which may affect tail protein phosphorylation and suppress sperm hyperactivated motility ^56,72^. Regardless, our data indicate that the UPS is necessary for sperm release induced by progesterone, cBiMPS, 4-AP, and procaine.

The in vivo trigger of sperm hyperactivation is uncertain. Although it does not activate CatSper in mouse sperm ^21^, progesterone is a strong candidate to activate sperm release in humans and some other species because it binds to a sperm membrane receptor, increases Ca^2+^ influx, and affects sperm motility ^33,34,36,37,59,73–75^. The source of progesterone could be follicular fluid from the ovulated oocyte, secretion from the ovulated oocyte-cumulus complex, or production from peri-ovulatory follicles and transport through counter-current mechanism ^76,77^. There is some doubt though that agents produced by the oocyte or cumulus cells could reach the isthmus at concentrations adequate to affect spermatozoa within sperm reservoir ^32^.

Assessment of hyperactivated motility has most often been performed on video recordings of motility by manual analysis of patterns of individual sperm^78^ or by CASA. Manual analysis of sperm trajectories is relatively subjective. Automated methods of measurement such as CASA, although more objective, suffer from the problem that hyperactivation cannot be defined as a “one size fits all” ^79^ due to species-specific differences in the sperm flagellum morphology and motility patterns ^80^. Thus, CASA of porcine sperm, when used to determine the percentage of hyperactivated sperm, can sometimes yield unclear results ^81^. In these experiments, hyperactivation was directly visualized using the minimum intensity projection of high-speed video microscopy data, which allowed the observation of tail motion in a single image. The sperm head was frequently immobilized when tail beat patterns were assessed. In that situation, asymmetrical tail beating resulted in the sperm pivoting around the head. The amount of rotation could be estimated objectively, and the rotation was an indication of beat asymmetry and “full-type” hyperactivation. This approach proved very useful as a more objective measurement of sperm hyperactivation.

In summary, our results indicate that complete porcine sperm hyperactivation can be induced independently of capacitation by activation of the cAMP and CatSper/Ca^2+^ systems. Further, complete hyperactivation is sufficient to induce sperm release from oviduct glycans. Sperm release is dependent on CatSper and the function of the UPS, suggesting that both the additional force generated by sperm hyperactivation ^49,82^ and sperm membrane protein degradation have important roles in sperm release.

## Supplementary Information

Supplementary Information 1.

Supplementary Video 1.

Supplementary Video 2.

Supplementary Video 3.

Supplementary Video 4.

Supplementary Information 2.

Supplementary Information 3.

## References

[1] Tung CK, Suarez SS (2021). Co-adaptation of physical attributes of the mammalian female reproductive tract and sperm to facilitate fertilization. Cells.

[2] Holt WV (2011). Mechanisms of sperm storage in the female reproductive tract: an interspecies comparison. Reprod. Domest. Anim..

[3] Talevi R, Gualtieri R (2010). Molecules involved in sperm-oviduct adhesion and release. Theriogenology.

[4] Silva E, Frost D, Li L, Bovin N, Miller DJ (2017). Lactadherin is a candidate oviduct Lewis X trisaccharide receptor on porcine spermatozoa. Andrology.

[5] Kadirvel G (2012). Porcine sperm bind to specific 6-sialylated biantennary glycans to form the oviduct reservoir. Biol. Reprod..

[6] Machado SA (2014). LewisX-containing glycans on the porcine oviductal epithelium contribute to formation of the sperm reservoir. Biol. Reprod..

[7] Miller DJ (2015). Regulation of sperm function by oviduct fluid and the epithelium: Insight into the role of glycans. Reprod. Domest. Anim..

[8] Machado SA, Sharif M, Kadirvel G, Bovin N, Miller DJ (2020). Adhesion to oviduct glycans regulates porcine sperm Ca2+ influx and viability. PLoS ONE.

[9] Machado SA, Sharif M, Wang H, Bovin N, Miller DJ (2019). Release of porcine sperm from oviduct cells is stimulated by progesterone and requires CatSper. Sci Rep.

[10] Ardon F (2016). Dynamics of bovine sperm interaction with epithelium differ between oviductal isthmus and ampulla. Biol. Reprod..

[11] DasGupta S, Mills CL, Fraser LR (1993). Ca(2+)-related changes in the capacitation state of human spermatozoa assessed by a chlortetracycline fluorescence assay. J. Reprod. Fertil..

[12] Fraser LR, Abeydeera LR, Niwa K (1995). Ca(2+)-regulating mechanisms that modulate bull sperm capacitation and acrosomal exocytosis as determined by chlortetracycline analysis. Mol. Reprod. Dev..

[13] Sharif M, Kerns K, Sutovsky P, Bovin N, Miller DJ (2021). Progesterone Induces porcine sperm release from oviduct glycans in a proteasome-dependent manner. Reproduction.

[14] Buffone MG, Hirohashi N, Gerton GL (2014). Unresolved questions concerning mammalian sperm acrosomal exocytosis. Biol. Reprod..

[15] Harayama H (2018). Flagellar hyperactivation of bull and boar spermatozoa. Reprod. Med. Biol..

[16] Chang MC (1984). The meaning of sperm capacitation A historical perspective. J Androl.

[17] Visconti PE (2009). Understanding the molecular basis of sperm capacitation through kinase design. Proc. Natl. Acad. Sci. U.S.A.

[18] Kojima A (2015). Roles of extracellular Ca(2+) in the occurrence of full-type hyperactivation in boar ejaculated spermatozoa pre-incubated to induce the cAMP-triggered events. Andrology.

[19] Otsuka N, Harayama H (2017). Characterization of extracellular Ca(2+) -dependent full-type hyperactivation in ejaculated boar spermatozoa preincubated with a cAMP analog. Mol. Reprod. Dev..

[20] Ho HC, Suarez SS (2001). Hyperactivation of mammalian spermatozoa: function and regulation. Reproduction.

[21] Lishko PV (2012). The control of male fertility by spermatozoan ion channels. Annu. Rev. Physiol..

[22] Suarez SS (2008). Control of hyperactivation in sperm. Hum Reprod. Update.

[23] Sun XH (2017). The Catsper channel and its roles in male fertility: A systematic review. Reprod. Biol. Endocrinol..

[24] Ren D (2001). A sperm ion channel required for sperm motility and male fertility. Nature.

[25] Carlson AE (2009). Pharmacological targeting of native CatSper channels reveals a required role in maintenance of sperm hyperactivation. PLoS ONE.

[26] Clapham DE, Hulse RE (2021). Sperm CatSper ion channel swims into sharper focus. Nature.

[27] Ho K, Wolff CA, Suarez SS (2009). CatSper-null mutant spermatozoa are unable to ascend beyond the oviductal reservoir. Reprod. Fertil. Dev..

[28] Gahlay GK, Rajput N (2020). The enigmatic sperm proteins in mammalian fertilization: an overviewdagger. Biol. Reprod..

[29] Carlson AE (2003). CatSper1 required for evoked Ca2+ entry and control of flagellar function in sperm. Proc. Natl. Acad. Sci. U.S.A.

[30] Olson SD, Fauci LJ, Suarez SS (2011). Mathematical modeling of calcium signaling during sperm hyperactivation. Mol. Hum. Reprod..

[31] Liu J, Xia J, Cho KH, Clapham DE, Ren D (2007). CatSperbeta, a novel transmembrane protein in the CatSper channel complex. J. Biol. Chem..

[32] Chang H, Suarez SS (2010). Rethinking the relationship between hyperactivation and chemotaxis in mammalian sperm. Biol. Reprod..

[33] Lishko PV, Botchkina IL, Kirichok Y (2011). Progesterone activates the principal Ca2+ channel of human sperm. Nature.

[34] Sagare-Patil V (2012). Differential concentration and time dependent effects of progesterone on kinase activity, hyperactivation and acrosome reaction in human spermatozoa. Int. J. Androl..

[35] Alasmari W (2013). The clinical significance of calcium-signalling pathways mediating human sperm hyperactivation. Hum. Reprod..

[36] Strunker T (2011). The CatSper channel mediates progesterone-induced Ca2+ influx in human sperm. Nature.

[37] Tamburrino L, Marchiani S, Muratori M, Luconi M, Baldi E (2020). Progesterone, spermatozoa and reproduction: An updated review. Mol. Cell Endocrinol..

[38] Chung JJ (2014). Structurally distinct Ca(2+) signaling domains of sperm flagella orchestrate tyrosine phosphorylation and motility. Cell.

[39] Miller MR (2016). Unconventional endocannabinoid signaling governs sperm activation via the sex hormone progesterone. Science.

[40] Achikanu C, Pendekanti V, Teague R, Publicover S (2018). Effects of pH manipulation, CatSper stimulation and Ca2+-store mobilization on [Ca2+]i and behaviour of human sperm. Hum. Reprod..

[41] Nolan MA (2004). Sperm-specific protein kinase A catalytic subunit Calpha2 orchestrates cAMP signaling for male fertility. Proc. Natl. Acad. Sci. U.S.A.

[42] Buffone MG, Wertheimer EV, Visconti PE, Krapf D (1842). Central role of soluble adenylyl cyclase and cAMP in sperm physiology. Biochim. Biophys. Acta..

[43] Tateno H (2013). Ca2+ ionophore A23187 can make mouse spermatozoa capable of fertilizing in vitro without activation of cAMP-dependent phosphorylation pathways. Proc. Natl. Acad. Sci. U.S.A.

[44] Alonso CAI (2017). Extracellular cAMP activates molecular signalling pathways associated with sperm capacitation in bovines. Mol. Hum. Reprod..

[45] Leahy T, Gadella BM (2011). Sperm surface changes and physiological consequences induced by sperm handling and storage. Reproduction.

[46] Sostaric E, van de Lest CH, Colenbrander B, Gadella BM (2005). Dynamics of carbohydrate affinities at the cell surface of capacitating bovine sperm cells. Biol. Reprod..

[47] Chang H, Suarez SS (2012). Unexpected flagellar movement patterns and epithelial binding behavior of mouse sperm in the oviduct. Biol. Reprod..

[48] Simons J, Olson S, Cortez R, Fauci L (2014). The dynamics of sperm detachment from epithelium in a coupled fluid-biochemical model of hyperactivated motility. J. Theor. Biol..

[49] Chang H, Suarez SS (2011). Two distinct Ca(2+) signaling pathways modulate sperm flagellar beating patterns in mice. Biol. Reprod..

[50] Arai Y, Sakase M, Fukushima M, Harayama H (2019). Identification of isoforms of calyculin A-sensitive protein phosphatases which suppress full-type hyperactivation in bull ejaculated spermatozoa. Theriogenology.

[51] Mizuno Y (2015). Distinct segment-specific functions of calyculin A-sensitive protein phosphatases in the regulation of cAMP-triggered events in ejaculated bull spermatozoa. Mol. Reprod. Dev..

[52] Vadnais ML, Galantino-Homer HL, Althouse GC (2007). Current concepts of molecular events during bovine and porcine spermatozoa capacitation. Arch. Androl..

[53] Kerns K, Morales P, Sutovsky P (2016). Regulation of sperm capacitation by the 26S proteasome: An emerging new paradigm in spermatology. Biol. Reprod..

[54] Zigo M, Jonakova V, Manaskova-Postlerova P, Kerns K, Sutovsky P (2019). Ubiquitin-proteasome system participates in the de-aggregation of spermadhesins and DQH protein during boar sperm capacitation. Reproduction.

[55] Taraschi A (2020). Two-player game in a complex landscape: 26S proteasome, PKA, and intracellular calcium concentration modulate mammalian sperm capacitation by creating an integrated dialogue-A computational analysis. Int. J. Mol. Sci..

[56] Kerns K, Zigo M, Drobnis EZ, Sutovsky M, Sutovsky P (2018). Zinc ion flux during mammalian sperm capacitation. Nat. Commun..

[57] Bovin NV (1993). Synthesis of polymeric neoglycoconjugates based on N-substituted polyacrylamides. Glycoconj. J..

[58] Machado SA (2014). Lewis X-containing glycans on the porcine oviductal epithelium contribute to formation of the sperm reservoir. Biol. Reprod..

[59] Mannowetz N, Miller MR, Lishko PV (2017). Regulation of the sperm calcium channel CatSper by endogenous steroids and plant triterpenoids. Proc. Natl. Acad. Sci. U.S.A.

[60] Chavez JC (2018). Acrosomal alkalization triggers Ca(2+) release and acrosome reaction in mammalian spermatozoa. J. Cell Physiol..

[61] Sutovsky P (2011). Sperm proteasome and fertilization. Reproduction.

[62] Zigo M, Kerns K, Sutovsky M, Sutovsky P (2018). Modifications of the 26S proteasome during boar sperm capacitation. Cell Tissue Res..

[63] Smith TT, Yanagimachi R (1991). Attachment and release of spermatozoa from the caudal isthmus of the hamster oviduct. J. Reprod. Fertil..

[64] Ded L, Hwang JY, Miki K, Shi HF, Chung JJ (2020). 3D in situ imaging of the female reproductive tract reveals molecular signatures of fertilizing spermatozoa in mice. Elife.

[65] Wang T (2020). The Ca(2+) channel CatSper is not activated by cAMP/PKA signaling but directly affected by chemicals used to probe the action of cAMP and PKA. J. Biol. Chem..

[66] Orta G (2018). CatSper channels are regulated by protein kinase A. J. Biol. Chem..

[67] Navarro B, Kirichok Y, Clapham DE (2007). KSper, a pH-sensitive K+ current that controls sperm membrane potential. Proc. Natl. Acad. Sci. U.S.A..

[68] Williams HL (2015). Specific loss of CatSper function is sufficient to compromise fertilizing capacity of human spermatozoa. Hum. Reprod..

[69] Wu ZZ, Li DP, Chen SR, Pan HL (2009). Aminopyridines potentiate synaptic and neuromuscular transmission by targeting the voltage-activated calcium channel beta subunit. J. Biol. Chem..

[70] Zapata-Carmona H (2019). The activation of the chymotrypsin-like activity of the proteasome is regulated by soluble adenyl cyclase/cAMP/protein kinase A pathway and required for human sperm capacitation. Mol. Hum. Reprod..

[71] Miles EL (2013). Transgenic pig carrying green fluorescent proteasomes. Proc. Natl. Acad. Sci. U.S.A.

[72] Kerns K, Zigo M, Sutovsky P (2018). Zinc: A necessary ion for mammalian sperm fertilization competency. Int. J. Mol. Sci..

[73] Calogero AE (2000). Effects of progesterone on sperm function: mechanisms of action. Hum. Reprod..

[74] Romero-Aguirregomezcorta J, Cronin S, Donnellan E, Fair S (2019). Progesterone induces the release of bull spermatozoa from oviductal epithelial cells. Reprod. Fertil. Dev..

[75] Uhler ML, Leungt A, Chan SYW, Wang C (1992). Direct effects of progesterone and antiprogesterone on human sperm hyperactivated motility and acrosome reaction**Presented in part at the 39th Annual Meeting of the Society of Gynecologic Investigation, San Antonio, Texas, March 18 to 21, 1992. Fertil. Steril..

[76] Hunter RH, Cook B, Poyser NL (1983). Regulation of oviduct function in pigs by local transfer of ovarian steroids and prostaglandins: A mechanism to influence sperm transport. Eur. J. Obstet. Gynecol. Reprod. Biol..

[77] Novak S, Almeida FR, Cosgrove JR, Dixon WT, Foxcroft GR (2003). Effect of pre- and postmating nutritional manipulation on plasma progesterone, blastocyst development, and the oviductal environment during early pregnancy in gilts. J. Anim. Sci..

[78] Marquez B, Suarez SS (2004). Different signaling pathways in bovine sperm regulate capacitation and hyperactivation. Biol. Reprod..

[79] Bernecic NC, Gadella BM, Leahy T, de Graaf SP (2019). Novel methods to detect capacitation-related changes in spermatozoa. Theriogenology.

[80] Kay VJ, Robertson L (1998). Hyperactivated motility of human spermatozoa: A review of physiological function and application in assisted reproduction. Hum. Reprod. Update.

[81] Daigneault BW (2014). Novel and traditional traits of frozen-thawed porcine sperm related to in vitro fertilization success. Theriogenology.

[82] Curtis MP, Kirkman-Brown JC, Connolly TJ, Gaffney EA (2012). Modelling a tethered mammalian sperm cell undergoing hyperactivation. J. Theor. Biol..

