# Supplementary figures and images for: Hyperactivation is sufficient to release porcine sperm from immobilized oviduct glycans
Source: Sci Rep. 2022 Apr 19;12:6446. doi: 10.1038/s41598-022-10390-x (PMC9019019; doi:10.1038/s41598-022-10390-x)

**Streptavidin-Sepharose Beads**

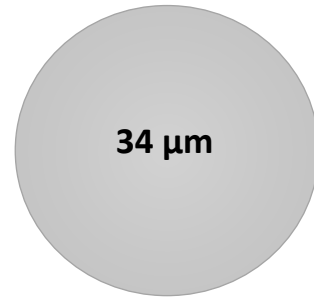

**Biotinylated Glycans**

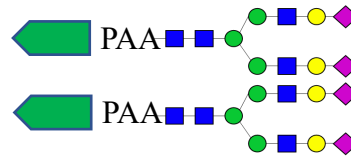

**Glyco-Bead**

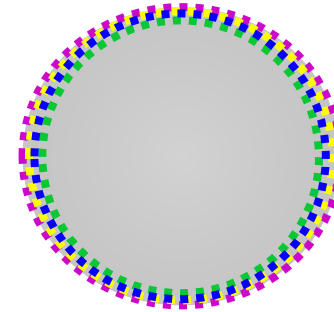

**Sperm bound to Glyco-Bead**

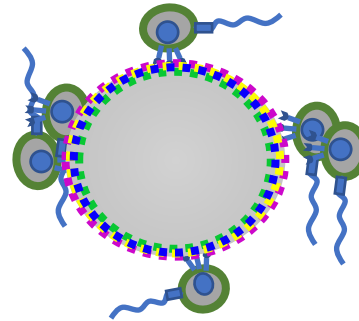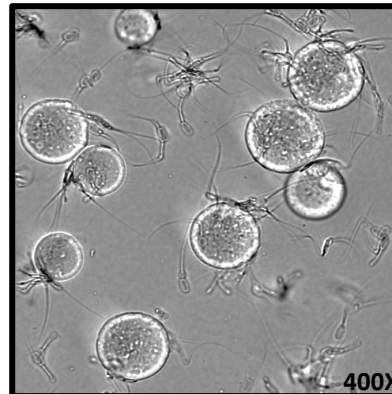

Supplement: Supplementary file 1 — Supplementary Information 1. [file 41598_2022_10390_MOESM1_ESM.pdf]
