# Supplementary material for: Hyperactivation is sufficient to release porcine sperm from immobilized oviduct glycans
Source: Sci Rep. 2022 Apr 19;12:6446. doi: 10.1038/s41598-022-10390-x (PMC9019019; doi:10.1038/s41598-022-10390-x)
Supplement: Supplementary file 7 — Supplementary Information 3. [file 41598_2022_10390_MOESM7_ESM.pdf]

Supplementary Figures

Supplementary Figure S1. Diagram of production of glycans covalently coupled to a biotinylated 20 kDa polyacrylamide chain that was, in turn, attached to streptavidin-coated beads. Beads to which $suLe^{X}$ was attached bound an average of 7 sperm/bead.

Supplementary Movie S1. Movie of sperm with symmetrical motility. This movie was taken 30 min after the addition of vehicle control.

Supplementary Movie S2. Movie of sperm with symmetrical motility. This movie was taken 30 min after the addition of vehicle control. This is the same movie as in Supplementary Movie 2 shown at 1/5 speed so that the motility pattern can be observed more easily.

Supplementary Movie S3. Movie of two fully hyperactivated sperm with asymmetrical motility. This movie was taken 30 min after the addition of 4-AP.

Supplementary Movie S4. Movie of two fully hyperactivated sperm with asymmetrical motility. This movie was taken 30 min after the addition of 4-AP. This is the same movie as in Supplementary Movie 3 shown at 1/5 speed so that the motility pattern can be observed more easily.

Supplementary Table 1. The concentrations of hyperactivation inducers and inhibitors that were used and the references of papers reporting an evaluation of these concentrations.
